# Special foods and local herbs used to enhance breastmilk production in Ghana: rate of use and beliefs of efficacy

**DOI:** 10.1186/s13006-020-00339-z

**Published:** 2020-11-16

**Authors:** Zakari Ali, Mohammed Bukari, Anita Mwinisonaam, Abdul-Latif Abdul-Rahaman, Abdul-Razak Abizari

**Affiliations:** 1 Department of Nutritional Sciences, School of Allied Health Sciences, University for Development Studies, P.O. Box 1883, Tamale, Ghana; 2 Nutrition Theme, MRC Unit The Gambia at the London School of Hygiene & Tropical Medicine, Atlantic Boulevard, Fajara, P.O. Box 273, Banjul, The Gambia

**Keywords:** Breastfeeding, Breastmilk, Lactogogues, Galactogogue, Lactation, Ghana

## Abstract

**Background:**

Inadequate breastmilk production is one of the key factors associated with suboptimal breastfeeding. In most local African homes, special herbs and some food items are commonly used to promote breastmilk production (known as lactogogue/galactogogue). We describe the use and characterize the herbs and food items used to promote breastmilk production in two regions of Ghana.

**Methods:**

We conducted a cross-sectional study in 2018 involving 402 lactating mothers. The range of foods used as lactogogues was obtained from 20 participants through focus group discussions. Quantitative data on demographics, lactogogue use and feeding practices were obtained through questionnaire administration.

**Results:**

The mean age of women was 29.2 years and children were 10 months. Breastmilk production problems were low (22.4%) and the majority of lactating mothers felt they had adequate breastmilk (70.4%) but awareness about lactogogues was widespread in both regions (88.8%) and highest in the Brong-Ahafo region (90.0%). Information about lactogogues was mainly from grandparents (24.6%), parents (31.6), health facilities (16.5%) and friends (12.8%), while the media had little influence (< 1%). The majority of the mothers used lactogogues to enhance breastmilk production (67.7%), and a quarter of them used lactogogues because of their tradition (25.2%). Prevalence of lactogogue use was 83.8%, lactogogues were prepared separate from household meals (59.4%) and consumed one to three times a day (89.6%). Users felt the effectiveness within 24 h of use (98.5%). The most common lactogogues included; groundnut/peanut soup prepared with *Bra leaves* (*Hibiscus sabdariffa*), hot black tea, *Werewere/Agushi (Citrulus colocynthis)* prepared with *Bra leaves,* and *Abemudro (a polyherbal formulation).* Only 13.2% of lactating mothers also used lactogogues during pregnancy.

**Conclusions:**

Special foods and selected herbs are widely used to enhance breastmilk production in Ghana and constitute an important part of the diet of lactating mothers. These results could contribute to understanding breastfeeding behaviours and stimulate further research into evaluating the safety and scientific efficacy of these products in Ghana.

## Background

Globally, an estimated 12% of mortality in children under five years of age is attributable to suboptimal breastfeeding practices [1]. Breastfeeding is considered the most important source of nutrition in the first year of life for both term and preterm infants because of its well-known positive effects on short and long-term outcomes [1]. In 2012, the World Health Assembly endorsed a comprehensive implementation plan on maternal, infant and young child nutrition, which specified six global nutrition targets to be achieved by 2025. Among others, the Resolution seeks to increase rates of exclusive breastfeeding among infants (< 6 months) by at least 50% [2]. Eight countries are on track to meet four targets and three of these countries are African. Ghana is on track to meet three of the targets along with 12 other countries and even though the proportion of under-five children who are exclusively breastfed in Ghana (52.1%) is well above the west African average (32.5%), progress has stalled in recent years [3].

Exclusive breastfeeding and appropriate complementary feeding are key to the maintenance of the nutritional wellbeing of children. In fact, promotion of optimal breastfeeding practices is one of the most effective interventions to prevent deaths in children under five years worldwide [4]. Breastfeeding is also associated with reduced risk of childhood infections and obesity, and long term benefits may include protection against the development of type-2 diabetes and other non-communicable diseases [5]. Some studies also point to benefits in schooling years and performance [6, 7].

Lactation is a complex physiological mechanism involving hormonal, physical and emotional factors. Inadequate breastmilk production is among the key factors that increase the rate of suboptimal breastfeeding among lactating mothers [8, 9]. Even though perceived insufficient breastmilk supply have been reported [10], the problem of insufficient breastmilk production persists. Different approaches to enhance breastmilk production have been explored including medication, herbal preparations and some food substances [11].

Lactogogues (or galactogogues) are substances which could be used to assist initiation, maintenance, or augmentation of the rate of maternal milk synthesis [12]. They include both pharmaceutical and herbal or food-based preparations. The World Health Organization has mentioned its usage in stimulating increased lactation in exceptionally difficult circumstances; emphasis has only been laid on the use of pharmacological substances such as domperidone and metoclopramide with supporting evidence of effectiveness [13]. The GP Infant Feeding Network (UK), has also recommended its prescription in only exceptional circumstances [14]. The evidence base for pharmaceutical lactogogues such as domperidone and metoclopramide [15] is stronger than it is for herbal and food-based preparations where safety and concerns around insufficient study sizes have been raised [16]. While special herbs and food substances continue to be widely used in many settings around the world to enhance breastmilk production for centuries, evidence regarding their benefits or harms is largely absent.

In most local African homes, herbs and special foods are widely used in the diets of lactating mothers to enhance breastmilk production and in many cases, women without apparent milk production also use them as a customary practice. However, these practices are largely undocumented and their perceived effectiveness unknown. We aimed to describe these practices among lactating mothers in two regions of Ghana. The results could help in understanding the extent of usage and stimulate further research into the safety and scientific efficacy of common foods and herbs used as lactogogues in Ghana.

## Methods

### Study area

The study was conducted in two regions of Ghana, chosen to represent the northern and southern parts of Ghana and as much as possible reflect the diversity in cultures across the country. The then Brong-Ahafo region which has recently been further divided into three (Brong-Ahafo; Bono East; and Ahafo regions) was chosen to represent southern Ghana while the Northern region also divided into three (Northern; Savannah; and North east regions) was chosen to represent the northern part of the country.

The Brong-Ahafo region lies in the forest zone and is a major cocoa and timber producing area. The region had a population of 1,815,408 in 2010; recent estimates from the regional health directorate indicates that 181,931 children are under five years old [17], the predominant ethnic group in the region is Akan, except in Sene where the Guans predominate. Christianity has the largest following (71.0%), followed by Islam (16.1%) and traditional religion (4.6%). The literacy rate is 41.7% who can read and write English and a Ghanaian language [18, 19].

The Northern region had a census population of 2,479,461 in 2010. The total population of children 0–4 years was projected to be 3,624,270 by 2020. The region is much drier than the southern areas of Ghana due to its proximity to the Sahel and the Sahara. The vegetation consists predominantly of grassland, especially savanna with clusters of drought-resistant trees such as baobabs or acacias. Islam is the predominant religion (56.1%), followed by practitioners of the African traditional religion (21.3%), while Christianity (19.3%) and others make up the rest. The main ethnicities and languages spoken widely include Dagbani, Gonja and Mampruli. Literacy rate is low at 19.2% who can read and write English and a Ghanaian language [18, 20].

### Study design, population and sample

The study used a mixed method approach involving cross-sectional administration of questionnaires and focus group discussions (FGDs). The target population for the questionnaire administration was lactating women with children aged 0–23 months in the two regions. The focus group discussions targeted grandmothers and other elderly women who are involved in childcare and are involved in food preparation and decisions concerning lactating women as well as lactating women. We conducted one FGD in each region using a purposive sample of ten target women. Each FGD included a mix of grandmothers, lactating women and other elderly women and lasted between 45 and 60 min. The purpose of the FGD was to identify the range of common lactogogues used in each region. Each FGD was facilitated by one research assistant and another who noted the discussions which were also recorded. The research assistants spoke the local language of the study area which made facilitation and transcription easier. Data saturation was determined when no further views were forthcoming. Information from the FGDs were also used to inform questionnaire development for the potential lactogogues mothers used. This enabled us to have good descriptions of lactogogues respondents used because we had information on the possible ways (names) specific lactogogues were referred in the two regions. Lactating mothers who participated in the FGDs were excluded from the questionnaire (quantitative) part of the study. The quantitative survey involved the administration of questionnaires to elicit the specific lactogogues used, usage frequency, perceived effectiveness and demographic characteristics of 402 women with children 0–23 months in both regions (Northern region = 202, Brong-Ahafo region = 200). The initial questionnaire was pretested on five lactating women outside the study area and feedback used to improve the questionnaire development including sequence of questions and skips. Reliability was assessed by checking content of responses for consistency. The questionnaires were interviewer-administered by graduate nutrition research assistants.

We aimed to estimate a single proportion of lactogogue use and assumed a 50% prevalence, 5% margin of error and a resulting required 385 sample size. To account for possible questionnaire damage or significant missing data in some questionnaires, 402 participants were targeted in the two regions instead.

### Sampling technique

Purposive sampling was used to select one district from each region, districts were chosen to reflect both rural and urban mixes as possible. In the Northern region, the Nanton district was selected while the Asutifi south district was selected to represent the Brong-Ahafo region. Data collection for the questionnaire administration took place in households of a large community and each selected purposively to represent a mixture of peri-urban and rural participants from the districts. We selected the next large community if the required sample was not obtained in the initial selected community. All households with mothers who had children 0–23 months were identified in each selected community and used to construct a sampling frame for systematic random sampling. The required sample for each community was used to divide the sampling frame to give the sampling interval. A random number was chosen from the sampling interval to be the starting household; subsequent households were chosen by adding the sampling interval to the selected number. This was done until the required sample for each community was achieved, in households with more than one eligible woman, one was selected through simple random selection. All selected women agreed and consented to participate in the survey. In instances where the target women were out of the household, field staff re-visited for consent and interview. We purposively sampled FGDs participants from a different community in the selected district which was not selected for questionnaire administration.

### Analysis of data

We have presented simple statistics of the quantitative data as frequencies and percentages where variables are categorical; means and standard deviations are used in continuous variables. Range of foods identified in the FGDs were used to inform the questionnaire design for each region. The data from the FGDs were further thematically analyzed to identify emerging themes/groups of lactogogues used. As our aim is to describe the usage and beliefs of efficacy, we have not run further analytical statistics.

## Results

### Socio-demographic characteristics of sampled lactating women in Brong-Ahafo and northern regions

The overall sample for the study was 402 lactating mothers selected from the Brong-Ahafo (200) and Northern (202) regions of Ghana. Average age of lactating mothers was 29.2 ± 6.0 years, those in Northern region were slightly older (30.0 ± 5.2 years) than in the Brong-Ahafo region (28.3 ± 6.6 years) (Data not shown). The majority of the children were less than a year old (62.3%) and more than half were male (54.0%). More than eight in ten mothers were currently married (87.6%) and for women in the Northern region, they were all married. Nearly half (47.0%) had completed a basic education, were traders (38.1%) and had low/poor socioeconomic status (40.1%) (Table 1).

**Table 1:** Socio-demographic characteristics of respondents in Brong-Ahafo (*n* = 200) and Northern region (*n =* 202)

Characteristic	Brong-Ahafo ***n*** (%)	Northern ***n*** (%)	All ***n*** (%)
**Age of children (months)**			
0–6	63 (31.5)	67 (33.2)	130 (32.3)
7–12	59 (29.5)	63 (31.2)	122 (30.4)
> 12	78 (39.0)	72 (35.4)	150 (37.3)
**Sex of child**			
Male	113 (56.5)	107 (53.0)	220 (54.7)
Female	87 (43.5)	95 (47)	182 (45.3)
**Marital status**			
Married	150 (75.0)	202 (100)	352 (87.6)
Currently unmarried	50 (25.0)	0 (0.0)	50 (12.4)
**Mother’s educational status**			
Basic	141 (70.5)	48 (23.8)	189 (47.0)
Secondary/Tertiary	36 (18.0)	9 (4.4)	45 (11.2)
No formal education	23 (11.5)	145 (71.8)	168 (41.8)
**Occupation of mother**			
Trader/vendor	82 (41.0)	71 (35.2)	153 (38.1)
Agricultural work	40 (20.0)	91 (45.0)	131 (32.6)
Civil servant	5 (2.5)	3 (1.5)	8 (2.0)
Others	35 (17.5)	19 (9.4)	54 (13.4)
Currently not working	38 (19.0)	18 (8.9)	56 (13.9)
**Socio economic status (SES)**			
Poorest	48 (24.0)	38 (18.8)	86 (21.4)
Poor	39 (19.0)	36 (17.)	75 (18.7)
Medium	57 (38.0)	24 (11.8)	81 (20.1)
Wealthy	38 (19.0)	42 (20.8)	80 (19.9)
Wealthiest	18 (9.0)	62 (30.7)	80 (19.9)

### Dimensions of breastfeeding practice and lactogogue use in the two regions

Maternal concerns about breastmilk production problems were low (22.4%) and majority of them felt they had adequate breastmilk for their child (70.4%). However, awareness about lactogogues was widespread in both regions (88.8%) and highest in the Brong-Ahafo region (90.0%). The lactating mothers noted a number of sources for information about lactogogues; grandparents (24.6%) and parents (31.6) were the commonly cited sources among other sources including the health facility (16.5%) and friends (12.8%), but the media was not an important source of information about lactogogues (< 1%). Overall use of lactogogues was 83.8% and was about five percentage points higher in the Northern region than in the Brong-Ahafo region. Apart from using lactogogues to enhance breastmilk production (67.7%), tradition (25.2%), first birth (3%) and small breast size (2%) were common reasons for lactogogues use among lactating mothers. Lactogogues were usually prepared separate from household meals (59.4%), consumed one to three times in a day (89.6%) and their effectiveness felt within 24 h of use (98.5%). Lactogogue use in pregnancy was low (11.4%) and only 13.2% were aware of using them during pregnancy and for this group who used them, lactogogues were used mainly after the first three months of pregnancy (73.9%) (Table 2). The data also show that mothers who reported breastmilk production problems were more likely to use lactogogues compared to those who did not (90.0% vrs 82.1% respectively) (data not shown).

**Table 2:** Factors related to the use of lactogogues to enhance breastmilk production

Variable	***N***^a^	Brong-Ahafo region ***n*** (%)	***N***^a^	Northern region ***n*** (%)	All ***n*** (%)
**Do you have breastmilk production problem?**	200		202		
Yes		48 (24.0)		42 (20.8)	90 (22.4)
**Do you feel your breastmilk is adequate for your child?**	127		130		257
No		64 (50.4)		12 (9.2)	76 (29.6)
**Awareness on special foods/herbs used to enhance breastmilk production**	200		202		
Yes		180 (90.0)		177 (87.6)	357 (88.8)
**Where did you learn about the use of special foods/herbs for breastmilk production?**	180		178		
Grandparents		40 (22.2)		48 (27.0)	88 (24.6)
Health facility		28 (15.6)		31 (17.4)	59 (16.5)
Parents		81 (45.0)		32 (18.0)	113 (31.6)
Friends		10 (5.6)		36 (20.2)	46 (12.8)
Media		0 (0.0)		1 (0.6)	1 (0.3)
Others		21 (11.7)		30 (16.9)	51 (14.2)
**Do you use lactogogues?**	200		202		
Yes		163 (81.5)		174 (86.1)	337 (83.8)
**What is your main reason for using lactogogues?**	163		174		
Enhance breastmilk production		74 (45.4)		154 (88.5)	228 (67.7)
Tradition		80 (49.1)		5 (2.9)	85 (25.2)
This is my first birth		2 (1.2)		7 (4.0)	9 (2.7)
Breast size small		4 (2.5)		1 (0.6)	5 (1.5)
Others		3 (1.8)		7 (4.0)	10 (2.9)
**Number of times special foods/herbs taken in a day**	163		174		
1–3 times		156 (95.7)		146 (83.9)	302 (89.6)
Above 3 times		7 (4.3)		28 (16.1)	35 (10.4)
**How was special food/herb used?**	163		174		
Added to usual food		88 (54.0)		28 (16.1)	116 (34.4)
Prepared separately		54 (33.1)		146 (83.9)	200 (59.4
Others		21 (12.9)		0 (0.0)	21 (6.2)
**Time taken to experience the effect of the special food/ herb after utilization**	163		174		
Within 24 h		158 (96.9)		174 (100.0)	332 (98.5)
Within a week		5 (3.1)		0 (0.0)	5 (1.5)
**Awareness of lactogogue use during pregnancy**	200		202		
Yes		32 (16.0)		21 (18.4)	53 (13.2)
**Use of lactogogues during pregnancy**	200		202		
Yes		26 (13.0)		20 (9.9)	46 (11.4)
**Stage of pregnancy in which special food/herb was used**	26		20		
0–3 months		5 (19.2)		7 (35.0)	12 (26.1)
4–6 months		12 (46.2)		10 (50.0)	22 (47.8)
7–9 months		9 (34.6)		3 (15.0)	12 (26.1)

^a^*N* changes depending on relevant skips in the questions

### Common lactogogues used among lactating mothers

The focused group discussions identified the common foods and herbs used by lactating mothers to aid breastmilk production. These foods and herbs were categorized into three groups: herb related lactogogues including selected local vegetables, hot lactogogues (emphasis is on eaten hot) and groundnut related lactogogues. The herb/vegetable related lactogogues comprised of: *Abemudro (a polyherbal formulation)*, *Ayoyo/Jute leaves* (*Corchorus olitorius*), *Nkontonmire/cocoyam leaves (Colocasia esculenta), Kuuka/* dried baobab leaves (*Adansonia digitata*) and tiger nuts (*Cyperus esculentus*). Hot lactogogues included; hot millet porridge, hot black tea, hot salt petre porridge and hot *Tuo Zaafi* (made from maize flour). Groundnut/peanut related lactogogues consisted of mashed kenkey with groundnut, corn porridge with groundnut, *Aleefu* (*Amaranth sp.*) prepared with groundnut, *Bra leaves* (*Hibiscus sabdariffa*) soup with groundnut, *Werewere (Citrulus colocynthis)* soup; and groundnut only soup (Data not shown).

While some lactogogues were used widely in both regions, others were used mainly in one region and only sparingly in the other. For example, hot lactogogue use was higher in the Northern region (96.0%) than in the Brong-Ahafo region (5.6%). However, groundnut related lactogogues were 35 percentage points higher in the Brong-Ahafo region than in the Northern region. The herbs were also commonly used in the Brong-Ahafo region (43.5%) than in the Northern region (18.5%) (Table 3).

**Table 3:** Common lactogogues used among lactating mothers

Class of lactogogues	***N***^a^	Brong-Ahafo region ***n*** (%)	***N***^a^	Northern region ***n*** (%)	All ***n*** (%)
**Groundnut related lactogogue**	161		173		
Yes		129 (80.1)		78 (45.1)	207 (62.0)
**Herb related lactogogue**	161		173		
Yes		70 (43.5)		32 (18.5)	102 (30.5)
**Hot related lactogogue**^b^	161		173		
Yes		9 (5.6)		166 (96.0)	175 (52.4)
**Others**	161	88 (54.7)	173	17 (9.8)	105 (31.4)

*N*^a^ changes depending on relevant skips in the questions. ^b^Hot related lactogogue: prepared and eaten hot

## Discussion

This study sought to describe the food items and herbs used to enhance breastmilk production and the beliefs of their effectiveness in two regions of Ghana. The results show that special foods and selected herbs are widely used to enhance breastmilk production in Ghana and constitute an important part of the diet of lactating mothers. These special herbs and selected foods have been categorized into three groups; groundnut related lactogogues, herb related lactogogues and hot related/consumed lactogogues. There were few reported problems with breastmilk production and use of lactogogues in pregnancy.

Awareness of the use of selected foods and herbs to aid in breastmilk production was high in both regions, this is consistent with studies conducted in Malaysia and Australia [16, 21] where high knowledge on breastmilk production aids were reported. A study in the United States also reported high awareness on the use of special aids especially fenugreek to promote breastmilk production [11]. People with knowledge would have been aware of substances used to help in breastmilk production, in most cases awareness increases curiosity and the urge to learn more about these substances and hence the high level of knowledge reported.

The current study showed that most lactating women obtained information on selected foods and special herbs from the home mostly parents and grandparents. Contrary to the findings of the current study, some studies have reported the internet as a major source [11], for other studies, friends and family [21] and health service providers [22, 23] were the main sources of information on breastmilk production aids. Our results are consistent with the study by Budzynska et al. [24] in which most cultures transfer knowledge of breastmilk productions aids from generation to generation.

The high prevalence of lactogogue use among lactating women in both regions is consistent with earlier studies [25–27] but a little higher than reported in studies from some developed countries [11, 23, 28]. Wealth inequality could play a role in healthcare access and delivery, respondents in the current study (a developing country setting) where healthcare access is relatively low may be less likely to seek professional advice from healthcare professionals, thus resorting to information from the family on which foods and special herbs could be used to support breastmilk production. It is expected that access to professional advice might have increased the options available for respondents from developed countries and, therefore, the low usage of breastmilk production aids especially herbs that may have little scientific evidence of efficacy and less likely to be recommended by professionals. A few mothers reported having breastmilk production problems but the majority still used lactogogues, this finding may suggest that these foods and herbs are used largely for prophylactic purposes or as part of usual postpartum diet rather than as a lactogogue or for curative purposes. However, we are unable to determine how many mothers are reporting no breastmilk problems because they had used lactogogues as a prophylaxis.

The current study showed that almost all respondents from both regions reported experiencing the effect of lactogogues within 24 h of use. Similarly, other studies reported effectiveness of lactogogues within 24–72 h [12, 29]. However, some studies have reported increased breastmilk production following lactogogue intake without indication of the duration for which effectiveness was felt [21, 30]. This highlights that breastmilk production aids such as the selected foods and special foods are highly perceived to be effective, irrespective of the time taken for their effects to be felt but this finding does not indicate scientifically proven efficacy because they are participant perception of effectiveness.

The high use of groundnut/peanut in most of the lactogogues preparations, especially in the Brong-Ahafo region have been reported in other settings. The most popular lactogogue, fenugreek, used mostly in Europe and Asian countries [12, 31, 32] is from the pea family which includes groundnuts. Differences in which sub-type of the pea family is used may be geographically dependent on availability. Groundnuts are abundant in Ghana as opposed to fenugreek. There have been reports in the literature about their use and potential to increase breastmilk production [33]. They are protein rich foods which are believed to explain the lactogenic effect. The exact mechanisms are yet unknown but proposed to be through the action of special amino acids which are absorbed into the blood stream and synthesized to milk proteins, such as whey and casein in the mammary glands [34]. They could also exert a lactogenic effect by providing a source of fatty acids for breastmilk production [28].

Lactogogues taken hot (hot-related) were also widely used in the enhancement of breastmilk production mostly in the Northern region of Ghana especially hot black tea. This finding is in line with a study which identified black tea (*Camellia sinensis*) as a lactogogue even though caution was indicated due to the presence of caffeine in most commonly used brands in our setting which could cause irritability in the child if taken in excess [34]. The women particularly emphasized that the tea needed to be hot to be effective. Taking into consideration the temperature of the tea and the components of the tea, there are two schools of thought on the mechanism of action to promote breastmilk production in lactating women. The first being that tea contains polyphenols and flavonoids which are important components that promote breastmilk production through an interaction with dopamine receptors [35], and the second explanation being that, the hot temperature of the tea might cause a rapid increase in blood circulation or stimulate circulation for faster let down of milk. Mothers also said the hot tea ‘melts’ the contents of the breast for more milk production.

Herb and selected local vegetables were also a major lactogogue in this Ghanaian population. Their use was, however, relatively low compared to the other lactogogue types. The common herb used in the Brong-Ahafo region was *Abemudro* (a polyherbal formulation) mostly used to improve lactation, an earlier study in the eastern region of Ghana also reported its usage [26]. The use of lactogogues during pregnancy, though low, it was believed to prepare the breast for more milk production after birth.

Our results could be useful in targeted education for lactating women. Knowledge about the food items, herbs and beliefs of efficacy could also help healthcare professionals plan contents of educational programmes targeted at lactating mothers. This is important given the high belief of efficacy in these products without established scientific evidence of any effect. We can only reasonably speculate that because the herbs and foods identified in this study are commonly used in Ghana without apparent health harms may indicate some level of safety. However, as we show in this study, usage frequency and modes of preparation of these products may be different when they are destined for lactogogues than for normal usage. Therefore, there is the need for further research to determine their level of safety and efficacy as lactogogues in Ghana. In addition, high beliefs in the efficacy of local herbs and selected food items in promoting breastmilk production could mean that lactating women who have breastmilk production problems may not or will delay in seeking professional care, thus affecting effective breastfeeding. Therefore, it is important for healthcare professionals engaged in breastfeeding promotion to be aware of these practices and beliefs in order to identify and encourage those with problems to seek professional help.

This study has a number of strengths such as the use of FGDs to enhance a comprehensive profile of lactotogues used in the study areas. The use of two regions, chosen to represent the northern and southern parts of Ghana has enabled documentation of the different lactogogue use and practices. However, the study has some limitations worth noting. The exclusion of non-breastfeeding women likely introduced some bias in the lactogogue practices as experiences of current lactating women and non-lactating women may be different. However, inclusion of current lactating women possibly improved recall of lactogogue practices and beliefs. It is also possible that women who are more interested in using some foods and herbs as lactogogues during breastfeeding may recall their practices differently. In spite of these limitations, this study has provided ample light on the practices and beliefs of efficacy of special foods and local herbs used in promoting lactation in Ghana.

## Conclusions

Special foods and selected herbs are widely used to enhance breastmilk production in Ghana and constitute an important part of the diet of lactating mothers. These results could contribute to understanding breastfeeding behaviours and stimulate further research into evaluating the safety and scientific efficacy of these products in Ghana.

## Data Availability

The data supporting the conclusions of this article are included within the manuscript. The dataset could be obtained from the corresponding author upon reasonable request.
